# Utilization Patterns and Implementation Barriers in Adoption of Teledentistry Within Romanian Dental Practice

**DOI:** 10.3390/healthcare13233176

**Published:** 2025-12-04

**Authors:** Andrei Andronic, George Maniu, Victoria Birlutiu, Maria Popa

**Affiliations:** 1 Dental Medicine and Nursing Department, Faculty of Medicine, Lucian Blaga University, 550169 Sibiu, Romaniaamaria.popa@ulbsibiu.ro (M.P.); 2 Mathematics and Informatics Department, Research Center in Informatics and Information Technology, Faculty of Sciences, Lucian Blaga University, 550025 Sibiu, Romania; 3 Faculty of Medicine, Lucian Blaga University, 550169 Sibiu, Romania; 4 County Clinical Emergency Hospital, 550245 Sibiu, Romania

**Keywords:** teledentistry, digital health, dental practice, COVID-19, association rules, machine learning

## Abstract

**Background:** Teledentistry constitutes a key component of digital health, enabling remote oral healthcare delivery through information and communication technologies (ICT). The COVID-19 pandemic accelerated its global adoption; however, data regarding its implementation within Romanian dental practice remain limited. Understanding usage patterns, perceived benefits, and implementation barriers is essential for effective integration. **Objectives:** This study examined the adoption of teledentistry among dental practitioners in Sibiu County, Romania, identified its main applications, assessed professional perceptions, and explored barriers and their interrelations using association rule mining (ARM). **Methods:** A cross-sectional online survey was distributed in 2025 to all 630 registered dentists in Sibiu County. The questionnaire collected demographic data, usage patterns, perceived benefits, and barriers. A total of 197 valid responses were obtained (response rate: 31.2%). Descriptive statistics, Chi-square tests, and ARM were used to identify associations between usage contexts and recorded obstacles. **Results:** Overall, 44.6% of respondents reported using teledentistry tools, primarily for interdisciplinary consultations (29.4%), postoperative counseling (26.4%), and treatment monitoring (25.3%). The most frequently cited barriers were the inability to perform direct clinical examinations (71.5%), practitioner reluctance (37.1%), insufficient infrastructure (29.9%), and the lack of a clear legislative framework (27.4%). ARM revealed frequent co-occurrence patterns among these barriers. Practitioners with prior experience in teledentistry reported significantly higher perceived utility (58% vs. 22.1%) and greater interest in training (58% vs. 38.5%, *p* < 0.05). **Conclusions:** Teledentistry shows moderate but increasing adoption among Romanian dentists. Addressing current barriers, through legislative clarification, infrastructure development, targeted professional training, and public education, is essential for achieving sustainable integration into modern dental practice.

## 1. Introduction

Telehealth represents the most comprehensive and integrative paradigm within the spectrum of digital health services, encompassing an extensive array of health-related interventions and processes delivered remotely through the deployment of advanced information and communication technologies (ICT). This broad domain transcends the provision of direct clinical care—such as patient assessment, diagnostic evaluation, therapeutic management, and longitudinal monitoring—to also include critical supportive functions. These encompass professional education and continuous training of healthcare providers, administrative and operational management of health systems, biomedical and health services research, health promotion, as well as preventive and public health initiatives. By facilitating the seamless integration of these diverse activities, telehealth enhances the efficiency, accessibility, and quality of healthcare delivery across multiple settings [1,2,3].

Telemedicine represents a specialized subset within the broader framework of telehealth, characterized by the utilization of digital technologies and telecommunications infrastructure to remotely deliver clinical medical services remotely. This discipline primarily concentrates on the direct provision of healthcare interventions—including diagnostic assessments, therapeutic procedures, and longitudinal follow-up care—aimed at maintaining continuity of patient management outside conventional healthcare settings. By enabling synchronous or asynchronous interactions between healthcare providers and patients, telemedicine seeks to overcome geographical and logistical barriers, thereby promoting equitable access to high-quality medical care irrespective of patients’ physical location. This modality underscores the critical importance of preserving clinical efficacy and patient-centered communication within remote healthcare delivery systems [4,5].

Teledentistry, as a specialized domain within telemedicine, entails the application of information and communication technologies (ICT) to facilitate the remote provision of dental healthcare services. This encompasses the systematic assessment, diagnostic evaluation, and ongoing monitoring of patients presenting with oral and maxillofacial conditions. By leveraging digital platforms, teledentistry enables the extension of dental care beyond traditional clinical environments, thus enhancing accessibility, efficiency, and continuity of care for diverse patient populations [6,7].

Although teledentistry may be perceived as a relatively recent innovation, its conceptual foundations date back to 1989, with practical implementation occurring five years later under the auspices of the United States Army’s “Total Dental Access Project.” The positive outcomes demonstrated by this pioneering initiative catalyzed the adoption of teledentistry in other technologically advanced nations, including the United Kingdom, Australia, and Canada. Over subsequent decades, teledentistry has undergone substantial evolution, propelled by rapid advancements in digital technology and the progressive integration of information and communication technologies within the domain of dental medicine. This evolution has expanded the scope and efficacy of remote oral healthcare delivery, positioning teledentistry as an increasingly integral component of modern dental practice [8].

Teledentistry constitutes a progressive and transformative modality within dental healthcare, significantly enhancing patient accessibility to high-quality oral medical services, especially in geographically isolated, rural, or underserved populations. By harnessing advanced digital communication technologies, teledentistry effectively mitigates traditional geographic and logistical impediments, facilitating comprehensive remote patient evaluation, accurate diagnosis, and timely access to specialist consultations in complex clinical scenarios. This paradigm shift enables the delivery of dental care without necessitating the patient’s physical presence, thereby promoting equity in oral healthcare provision and optimizing resource allocation [2,9]. As a result, waiting times and intervention costs are significantly reduced [2,10] and disparities in access to healthcare services are also substantially diminished [9,11,12].

An additional significant advantage of teledentistry resides in its capacity to facilitate interdisciplinary consultations, thereby fostering enhanced diagnostic precision and contributing to the overall improvement of clinical care quality. This collaborative approach enables seamless communication and knowledge exchange among dental specialists and other healthcare professionals, ultimately supporting comprehensive patient management and optimizing therapeutic outcomes [12,13]. Moreover, the use of teledentistry supports continuous medical education by providing healthcare professionals [2,10] with easy access to professional training programs [14], while also promoting patient education through information and prevention initiatives [15,16]. Teledentistry has demonstrated considerable benefits for patient populations with special healthcare needs—including the elderly, individuals with disabilities, patients suffering from chronic medical conditions, and those residing in institutionalized environments—by facilitating access to essential dental care services that might otherwise be limited or inaccessible. This modality helps overcome physical, mobility, and logistical challenges, thereby promoting equitable oral healthcare delivery and improving overall patient outcomes within these vulnerable groups [13,14,17].

Despite ongoing technological advancements and the well-documented benefits associated with its implementation, teledentistry has not yet achieved widespread acceptance or garnered adequate interest among dental healthcare professionals. This limited engagement may be attributed to factors such as lack of familiarity with digital platforms, concerns regarding clinical efficacy, and perceived challenges in integrating telehealth solutions within conventional dental practice [11,18]. The primary barrier to the adoption of this technology remains the reluctance of healthcare professionals, largely justified by the inherent limitations of telemedicine—particularly the inability to perform direct clinical examinations and actual dental interventions [16,19,20]. Additionally, the lack of adequate infrastructure—including hardware and software equipment, specialized personnel, dedicated facilities, and a stable internet connection [13]—constitutes a significant obstacle, as it requires substantial investments and costs that have a major impact, especially in resource-limited areas [2,10,16]. Concurrently, the absence of clear legislative regulations generates uncertainties regarding safe and legally compliant practices [1,15,18]. Confidentiality and the safeguarding of personal health information constitute critical challenges within teledentistry, necessitating stringent compliance with regulatory frameworks governing informed consent, data privacy, and cybersecurity. Healthcare providers must ensure the secure handling, transmission, and storage of sensitive patient data to maintain trust and uphold ethical and legal standards [1,10,21].

In addition to these factors, patient reluctance [15,22], lack of digital literacy, and uncertainties regarding the optimal use of teledentistry in dental care contribute to the slow and fragmented adoption of this technology compared to other medical specialties [9].

The COVID-19 pandemic served as a critical catalyst for the rapid development and widespread adoption of teledentistry, expediting its evolution from a relatively niche and underutilized modality to an established component of dental healthcare delivery. Amid stringent social distancing mandates and severely restricted access to traditional in-person dental services, teledentistry emerged as an essential alternative for maintaining continuity of care [15,23], and a viable solution for providing urgent care, patient triage, and monitoring [18,20], thereby contributing to the reduction of exposure for both patients and healthcare personnel and consequently lowering the risk of virus transmission [2,24]. The deployment of teledentistry during the pandemic facilitated the uninterrupted provision of dental services within a framework that ensured patient and practitioner safety, simultaneously underscoring the technology’s potential to enhance accessibility and operational efficiency. Nonetheless, the crisis also brought to light significant limitations inherent to teledentistry, including the absence of comprehensive legislative frameworks, deficiencies in technical infrastructure, persistent professional hesitancy, and critical concerns regarding data privacy and security [2,20].

Therefore, the COVID-19 global health crisis has not only highlighted the transformative capabilities of teledentistry in maintaining continuity of care but has also emphasized the imperative for comprehensive systemic interventions. These interventions are essential to address existing infrastructural, regulatory, and professional barriers, thereby facilitating the sustainable and effective integration of teledentistry into standard dental practice.

In Romania, research focusing on the utilization and perception of telemedicine within the dental sector remains limited, with insufficient data available concerning the degree of adoption, specific challenges encountered, potential benefits, and domains of application. Against this backdrop, the present study seeks to systematically examine the prevalence and usage patterns of telemedicine among dental practitioners in Sibiu County, identify key barriers and facilitators influencing its implementation, and evaluate the impact of the COVID-19 pandemic on both the perception and uptake of this emerging technology.

The study’s specific objectives are as follows: (1) to quantify the extent of telemedicine integration in routine dental practice; (2) to delineate the principal clinical and non-clinical applications of telemedicine within dentistry; (3) to assess dental professionals’ attitudes towards the advantages and inherent limitations of telemedicine; (4) to identify the principal obstacles hindering telemedicine adoption; (5) to identify interdependencies between barriers and different contexts of utilization using a machine learning method (association rules) and (6) to investigate prospects for the future development and systematic incorporation of telemedicine technologies in dental care delivery. In alignment with these objectives, the study addresses the following research questions:

RQ1. To what extent has teledentistry been adopted and integrated into routine dental practice among dentists in Sibiu County, Romania?

RQ2. What are the main barriers and facilitating factors influencing the implementation and effective use of teledentistry in clinical dental settings?

RQ3. How do professional characteristics and prior experience with digital technologies influence dentists’ perceptions, utilization patterns, and willingness to engage in future teledentistry training programs?

## 2. Materials and Methods

To obtain the necessary data for this investigation, we conducted a cross-sectional survey using a non-probability convenience sampling method, in which data were collected through a structured online questionnaire developed and distributed via the Google Forms platform using targeted invitations. The survey was aimed specifically at dental practitioners operating within Sibiu County. The initial questionnaire design was informed by the study’s research objectives and was subsequently subjected to content validation by a panel of five dental experts affiliated with “Lucian Blaga” University, who provided critical feedback to enhance clarity, relevance, and thematic alignment. Their recommendations were incorporated into a revised version of the instrument.

Following this, the questionnaire underwent a methodological review by a professional statistician to ensure appropriateness of question format and analytical feasibility, culminating in a finalized instrument comprising 14 items organized into two primary sections (Appendix A). The first section block included six questions focused on capturing socio-demographic and professional characteristics of the participants (e.g., age, gender, years of experience, practice setting), while the second section block, containing eight questions, addressed aspects related to the utilization of teledentistry (U1: patient consultations, U2: monitoring of treatment progress, U3: postoperative patient counseling, U4: SARS-CoV-2 pandemic, U5: educational and preventive oral health, U6: consultations/ collaboration with other dental specialists), implementation challenges (B1: insufficient technical infrastructure, B2: patient hesitancy or reluctance to engage with telemedicine, B3: Inability to perform a direct, in-person clinical examination, B4: concerns related to data confidentiality and information security, B5: absence of explicit and comprehensive legislative frameworks governing telemedicine practice, B6: resistance to change among medical personnel), perceived benefits and limitations and the perceived necessity for targeted professional development programs.

The final questionnaire was electronically distributed via email to the entire cohort of dentists registered with the Sibiu College of Dental Physicians. Data collection occurred over a two-month interval between July and August 2025, during which two follow-up reminders were issued through WhatsApp to enhance response rates. Participation in the study was entirely voluntary, with informed consent obtained from all respondents prior to survey completion. Confidentiality and anonymity of all data were rigorously maintained. The survey yielded 197 completed responses from a sampling frame of 630 dental practitioners, resulting in a response rate of 31.2%.

To describe the study variables, we used number and percentages. Contingency tables, Chi-Square test, Fischer test, were used to identify whether there were significant differences between different use cases/barriers analyzed and questions related to practice settings, years of experience, utility of telemedicine in dental practice, perspectives, future directions.

Association rule mining (ARM) method was used to identify interdependencies between barriers and different contexts of utilization. The ARM technique, initially proposed by Agrawal et al. (1994) [25] in the context of market basket analysis for detecting and extracting products frequently purchased together, is a very popular machine learning method applied to gain useful insights into associations and hidden relationships of items, in medicine and also in other research areas [26,27,28,29,30,31].

In our context of analyzing situations of utilizations and barriers, associations rules can help uncover how different individual use cases or specific groups (combinations) of use cases are associated (have impact) with the occurrence of certain barrier(s).

The algorithm considers the set of items represented by utilizations areas and barriers and the “transaction set” represented by the respondent’s opinion on considered use cases and barriers. It works by identifying rules of the form “If A, then B”, where A and B are sets of items representing antecedent (or LHS—left-hand-side) and consequent (or RHS—right-hand-side) of the rule. The rules were generated and visualized using R-packages. We use a minimum support threshold of 1% (in order to not remove potentially interesting rules with lower support) and minimum confidence threshold of 50%. For the evaluation of the rules, we used metrics support, confidence and lift, to measure the frequency, reliability and strength of the item’s association.

## 3. Results

### 3.1. Demographic and Professional Profile of Participants

The demographic analysis of the study sample revealed that the highest proportion of respondents were dentists aged between 41 and 50 years (39.5%). In contrast, the lowest participation rate was noted among dentists over the age of 50, accounting for only 12.1% of respondents, which may reflect varying levels of engagement with digital health innovations or differing professional priorities within this cohort.

Regarding gender distribution, female practitioners predominated in the sample, representing 61.9% of the respondents. Professional qualification data showed that 33% of participants were dental specialists, with expertise in distinct branches of dentistry, while the majority, 67%, were general dental practitioners. With respect to professional tenure, 40.6% of respondents reported having between 11 and 20 years of clinical experience, suggesting a substantial segment of practitioners in mid to late career stages, while 26.4% possessed over 20 years of experience, representing a cohort with extensive clinical expertise and possibly more traditional practice approaches.

Analysis of the working environments indicated that a majority of respondents (51.8%) were employed in small-scale dental practices typically operated by one or two dentists. This practice setting often involves close patient-provider relationships but may face limitations in resources and technological infrastructure. Meanwhile, 24.9% of participants worked in medium-sized practices comprising three to five dentists, and 23.3% were affiliated with large dental clinics employing more than five practitioners, where more comprehensive organizational structures and potentially greater access to advanced technologies may exist.

### 3.2. Adoption and Utilization of Telemedicine in Dental Practice

A breakdown of telemedicine applications revealed diverse utilization patterns: 29.4% of respondents employed telemedicine to facilitate interdisciplinary collaboration and consultations with other dental specialists. Postoperative patient counseling via telemedicine was utilized by 26.4% of participants. Furthermore, 25.8% of dentists conducted patient consultations through digital platforms. Monitoring of treatment progress through telehealth modalities was reported by 25.3% of respondents.

The onset of the SARS-CoV-2 pandemic acted as a catalyst for telemedicine adoption, with 24.3% of dentists acknowledging an increased use of digital communication tools during this period. Conversely, telemedicine applications aimed at educational and preventive oral health initiatives were markedly less common, with only 16.2% of respondents indicating engagement in such activities.

### 3.3. Barriers to the Implementation of Telemedicine

The predominant barrier reported by dental practitioners hindering the integration of telemedicine into routine clinical practice was the inherent limitation posed by the inability to perform a direct, in-person clinical examination, which was identified by 71.5% of respondents. This obstacle underscores a fundamental challenge in reconciling the hands-on nature of dental care with remote modalities. Another salient impediment was the resistance or reluctance among medical personnel, reported by 37.06% of participants, which appears to stem from perceptions regarding the incongruity between the clinical demands of dentistry and the capabilities of remote assessment techniques.

Additional frequently cited challenges included insufficient technical infrastructure, reported by 29.9% of respondents, patient hesitancy or reluctance to engage with telemedicine (28.4%), and the absence of explicit and comprehensive legislative frameworks governing telemedicine practice (27.4%). Notably, concerns related to data confidentiality and information security constituted the least frequently mentioned barrier, with only 14.2% of participants indicating this as a significant issue.

The trends regarding utilization situations and barriers, for all study group and by dental clinic size or dental practitioner’s years of experience are presented in Table 1.

Thus, among the surveyed dental practitioners, 44.6% reported having engaged with telemedicine technologies in their clinical or professional activities, reflecting a moderate level of adoption within the study population. A comparative analysis revealed that respondents with prior experience in utilizing teledentistry demonstrated a heightened awareness and acknowledgment of implementation barriers relative to non-users. Despite this, the hierarchical order of perceived difficulties remained consistent across both cohorts, with the exception of medical personnel reluctance, which was significantly more pronounced among practitioners without telemedicine experience (*p* = 0.002). The comparative analysis of responses provided by general dentists and dental specialists did not reveal any statistically significant differences, with the exception of the area related to collaboration with other specialists (*p* = 0.009). In this regard, general dentists (23.48%) employ teledentistry for interdisciplinary consultations at a considerably lower rate than specialists (41.54%). No statistically significant differences were observed between general dentists and dental specialists in their perceptions of the barriers associated with implementing teledentistry.

### 3.4. Association Rule Mining Analysis

Using association rule mining (ARM) method was useful to identify the most frequent association of utilization situations and most frequent association of barriers (Table 2). The analysis of combinations of barriers stratified by respondents who used vs. respondents who did not used telemedicine technologies in their clinical or professional activities, shows that barrier B6 (resistance to change) is more frequently encountered, in combination with other barriers, in case of respondents who have not used telemedicine technologies.

**Figure 1:**
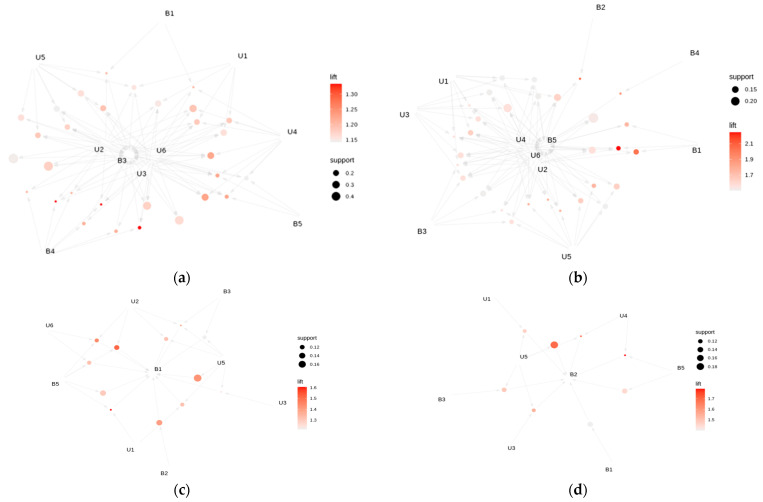
Graph-based visualization of association rules having as consequent the barrier: (**a**) B3; (**b**) B5; (**c**) B1; (**d**) B2. The nodes/vertices represent items or item sets and the rules are represented as directed edges between the items or item sets. Rules measure metrics lift and support are represented by color (intense red color for higher lift values) and size (bigger nodes/circles for higher support).

ARM method was used also to identify the association between the different types of usage situations and various barriers encountered. We analyze the association rules having individual barriers (single item) in the consequent of the rule (Figure 1).

The large number of rules with consequent barrier B3 and varying antecedents shows that B3 occur in a wide range of contexts defined by individual and different combinations of use cases (and in combination with other barriers). Use cases U2, U3 and U6 are among the most relevant when analyzing the context of the emergence of barrier B3 (Figure 1a). Another barrier which appears in a large number of rules having it as a consequence, is the barrier B5 (Figure 1b). The rules with high lift ({U2, U6, B1} → {B5} (lift 2.24), {U2, B1} → {B5} (lift 2.03), {U4, B2} → {B5} (lift 2.02)) show that B5 tends to occur not only in certain situations of utilization (U2, U4, U6), but often in contexts where other barriers are also present (such as B1 and B2). This suggests an interaction or frequent co-occurrence between B5 and these other barriers.

Barriers B1 (Figure 1c) and B2 (Figure 1d) appear as a consequence in a smaller number of rules in comparison with the case of barriers B3 and B5, indicating that only certain specific combinations of use cases and/or barriers lead to the occurrence of B1 and B2. Analyzing both the rules that have these barriers as a consequent, and the general set of association rules (sorted by the lift metric), it is observed that these barriers appears more frequently in the antecedent of rules with high lift, suggesting that B1 and B2 could play an important role as a factors contributing to the appearance of other barriers (such as barrier B5).

### 3.5. Respondents Perception of Utility

The distribution of perceptions regarding the utility of telemedicine in dental practice among the respondents revealed a predominance of neutral attitudes, with 31.4% adopting an indifferent or undecided stance on its clinical and practical value. Conversely, a combined 38.0% of participants expressed a positive evaluation of telemedicine, with 16.7% considering it useful and an additional 21.3% rating it as very useful. On the other hand, a minority of 7.6% of respondents perceived telemedicine as lacking any significant clinical or practical utility.

When stratified by prior experience with telemedicine, distinct differences emerged in perception. Among practitioners who had previously incorporated teledentistry into their professional activities, a notable majority of 58% affirmed its value, subdivided into 20.5% who regarded it as useful and 37.5% who considered it very useful. In contrast, respondents with no prior experience with teledentistry demonstrated considerably lower positive appraisal levels, with only 13.8% rating it as useful and 8.3% as very useful, cumulatively amounting to 22.1%.

### 3.6. Respondents Perspectives and Future Directions

Approximately 47.2% of the surveyed dental practitioners expressed a clear willingness and interest in participating in specialized training programs focused on the application and integration of telemedicine within dental practice. Notably, this interest was significantly more pronounced among practitioners with prior experience in teledentistry, with 58% of users indicating a desire for further professional development, compared to a comparatively lower 38.5% observed among non-users.

Concerning future prospects, 36% of respondents anticipate that telemedicine will assume a prominent and influential role in the evolution of dental practice. Furthermore, a proportion of 45.6% participants recognized that telemedicine and related digital technologies exerted a considerable impact on their clinical and professional activities during the SARS-CoV-2 pandemic, underscoring the transformative potential of these modalities in response to emergent healthcare challenges.

## 4. Discussion

This study offers a comprehensive analysis of the adoption rate and professional perceptions regarding telemedicine within the dental community of Sibiu County, Romania. The results underscore both the prospective advantages and the inherent challenges linked to the integration of telemedicine technologies within dental practice. Notably, 44.67% of the surveyed dental practitioners reported utilizing teledentistry services in varying capacities. This adoption rate significantly exceeds figures reported in comparable investigations, such as a 2021 study conducted in Saudi Arabia [24], which documented a 23.2% utilization rate, and a 2023 study from the United States, where teledentistry adoption was reported at 30% [22]. By contrast, a study conducted by Cheuk R. and colleagues in Ontario, Canada, in 2023 reported a higher utilization rate of teledentistry among Canadian dentists, reaching 49.3% [16]. Other studies conducted in France [17] and in the city of Chennai, India [14], report lower utilization rates, specifically 39.3% among French dental professionals and 35.6% among Indian practitioners.

The adoption of telemedicine demonstrates marked variability on a global scale, with reported utilization rates ranging from 23.2% in Saudi Arabia to as high as 49.3% in Canada. Within this spectrum, dental professionals in Romania appear to occupy an intermediate position, indicative of a moderate degree of integration of telemedicine technologies into routine dental practice. These discrepancies in adoption rates are likely multifactorial, influenced by a confluence of determinants such as the maturity of technological infrastructure, the extent of governmental policy support and regulatory frameworks, sociodemographic attributes of the patient population, as well as the recent transformative effects of the COVID-19 pandemic on healthcare delivery models [24,32,33].

The patterns and frequency of teledentistry utilization among the study cohort demonstrate a distinct inclination toward applications that circumvent direct physical interaction with patients. The highest reported uses included interdisciplinary collaboration with other specialists (29.4%), postoperative patient counseling (26.4%), and conducting consultations through digital platforms (25.8%). These results are consistent with international trends reported in the literature, such as those documented by Niknam et al. (2022) [20] and Buhaisi et al. (2024) [34], which identify teledentistry as predominantly deployed for primary consultations, specialist interdisciplinary communication, and longitudinal treatment monitoring. This preference underscores the modality’s role as an adjunctive tool facilitating communication and continuity of care while mitigating the constraints associated with face-to-face encounters [20,34].

Treatment monitoring (25.3%) and patient communication during the COVID-19 pandemic (24.3%) further emphasize the critical role of teledentistry in preserving continuity of care and sustaining the dentist–patient relationship amidst enforced social distancing measures. Supporting this observation, Hung et al. [18] reported that widely accessible commercial communication platforms, including WhatsApp, Skype, and Zoom, were predominantly utilized for triage and appointment management during the pandemic. These tools effectively minimized the need for in-person visits, enabling remote consultations and contributing to the reduction of potential viral transmission within dental settings [18].

A noteworthy finding from our study is the relatively low utilization of teledentistry for educational and preventive purposes, reported by only 16.2% of respondents. This underrepresentation signals a significant gap and an underexploited potential within the domain of oral public health. Similar concerns have been articulated by Nascimento da Silva et al. [35], who emphasize the critical need to integrate educational and preventive oral health services into digital health platforms to enhance population-wide health outcomes and promote proactive dental care [10,15].

Physicians who most often cited the lack of direct clinical examinations as a major barrier were primarily those who used teledentistry for purposes such as interdisciplinary collaboration, treatment follow-up, and postoperative patient counseling. This pattern underscores the intrinsic clinical limitations of teledentistry—particularly the challenges associated with remote physical assessment—while simultaneously delineating its practical and effective domains of application within dental practice.

In response to the first research question our study revealed a moderate level of teledentistry adoption, with 44.6% of surveyed dentists reporting the use of teledentistry tools in their professional activity. The most frequent applications included interdisciplinary consultations (29.4%), postoperative counseling (26.4%), and treatment monitoring (25.3%). These findings position Romanian dentists at an intermediate stage of digital integration, comparable to international contexts such as France (39.3%) and Canada (49.3%), indicating both openness to innovation and persistent structural limitations.

The overall attitudinal profile of dentists in Sibiu County regarding telemedicine reflects a moderately positive disposition, with a combined 69.4% of respondents expressing neutral to favorable perception of its utility. The response distribution indicates a modest polarization of opinion; notably, 38% of participants rated telemedicine as either useful or very useful, reflecting an encouraging degree of openness to its integration into routine clinical workflows. Conversely, the substantial proportion of neutral responses (31.4%) may signify an ongoing evaluative process among dental professionals, who are still discerning the advantages and constraints of telemedicine in the context of the unique demands and characteristics of their clinical practice.

The observed discrepancy between the generally favorable attitudes toward telemedicine and its comparatively low rate of practical implementation is a phenomenon well-documented in the extant scientific literature. This incongruity underscores the presence of substantial barriers—be they technological, regulatory, infrastructural, or behavioral—that impede the widespread adoption and seamless integration of telemedicine technologies within routine dental practice [24,36,37].

Moreover, the favorable perception of teledentistry markedly intensifies among practitioners who have direct experience with its use, with approximately 58% of users rating it as useful or very useful. In contrast, only 22.1% of non-users share this positive appraisal, highlighting a significant disparity in attitudes based on practical engagement. This trend aligns with findings reported by Hung et al. [18], who documented that nearly 75% of dentists with prior teledentistry experience intend to sustain or increase their utilization of such services. These data underscore not only a positive professional perception but also a strong recognition of the clinical and operational benefits associated with the technology [18].

The findings of the present study elucidate multiple impediments to the widespread adoption of telemedicine within dental practice, predominantly attributable to the inherently interventional nature of dentistry and its dependence on direct physical examination. The foremost limitation identified was the inability to conduct comprehensive in-person clinical assessments, a concern reported by 71.5% of participants. This predominant barrier is consistent with observations documented in extant international research, underscoring the intrinsic challenges posed by remote modalities in a specialty reliant on tactile and visual intraoral evaluation [20,36,38].

Empirical studies conducted in comparable clinical and geographical contexts underscore that the absence of direct clinical examination—specifically through tactile inspection and palpation—can significantly compromise diagnostic accuracy. Consequently, the scope of telemedicine applications within dental practice remains largely constrained to functions such as patient triage, treatment monitoring, and counseling, rather than serving as a substitute for comprehensive diagnostic evaluation [39,40].

Similarly, reluctance among healthcare professionals—expressed as a perceived incongruity between the inherently hands-on nature of dental practice and the modalities of remote consultation, reported by 37% of respondents—serves to reinforce skepticism regarding the practical utility and effectiveness of telemedicine in dentistry. This apprehension may be closely associated with limited prior experience and insufficient professional training in the utilization of digital health technologies, a correlation that has also been documented in the literature [12]. Moreover, the systematic integration of telemedicine and digital health technologies into continuing professional development and medical education curricula represents a strategic imperative. Such initiatives are essential to foster higher acceptance among dental practitioners and to cultivate the requisite digital literacy and technical competencies necessary for effective implementation and utilization of telemedicine within clinical practice [6].

Another critical barrier identified by respondents is the insufficiency of technical infrastructure, reported by 29.9% of participants. This limitation exerts a disproportionately adverse effect on small-scale dental practices as well as those situated in rural or underserved regions. Deficiencies in access to advanced diagnostic and communication equipment, reliable high-speed internet connectivity, and secure digital platforms significantly compromise the efficacy and quality of remote clinician–patient interactions. Such infrastructural constraints are not unique to Romania but are also prevalent in other countries exhibiting comparable stages of technological development [17,36], thereby representing a widespread challenge to the equitable implementation of telemedicine in dentistry. Strengthening the IT infrastructure within the healthcare sector and ensuring stable internet connectivity represent fundamental prerequisites for the advancement of medical service digitalization. In this context, the development and implementation of dedicated governmental funding programs, supported by national resources or European funds, are recommended to assist small dental practices and those located in rural areas in the adoption and integration of new digital technologies.

Surprisingly, concerns related to data confidentiality and security elicited the lowest level of apprehension among respondents, being cited by only 14.2% of dentists. This finding diverges from prevailing global trends, where data protection issues consistently rank among the foremost challenges in the adoption and implementation of digital health technologies [10,36].

The comparatively diminished emphasis on these concerns by dental practitioners in Sibiu may reflect either an underestimation of the inherent risks associated with handling sensitive patient information in digital formats or a tendency to prioritize more immediate practical and operational barriers.

Within the broader international framework, data security is regarded as a fundamental component of digital healthcare provision. Regulatory frameworks such as the European Union’s General Data Protection Regulation (GDPR) or The Health Insurance Portability and Accountability Act (HIPPA) in the United States [41] mandate stringent standards for the safeguarding of personal health data. Compliance with these regulations requires healthcare providers to establish and maintain robust technical, administrative, and organizational measures to ensure the confidentiality, integrity, and security of patient information when employing telemedicine and other digital health solutions [34,42]. Possible solutions to strengthen the protection of patient’s medical data may include the implementation of secure digital platforms compliant with international standards such as GDPR or HIPAA, the integration of advanced authentication and access control mechanisms, the use of end-to-end encryption for data transmission, the assurance of secure data storage, and the systematic training of medical personnel in confidentiality and cybersecurity protocols.

Patient reluctance to engage with telemedicine emerged as a noteworthy barrier, reported by 28.4% of the surveyed dental practitioners. This hesitation may stem from several factors, including limited awareness or understanding of telemedicine services, concerns about the effectiveness of remote consultations, and a traditional preference for face-to-face interactions within the dental office setting. Such attitudes reflect the deeply ingrained expectations of direct personal contact in dental care, which is often perceived as essential for accurate diagnosis and treatment.

Nonetheless, existing research suggests that patient acceptance of telemedicine can be significantly enhanced through positive firsthand experiences and targeted educational initiatives. Studies have demonstrated that when patients are adequately informed about the benefits, safety, and convenience of telemedicine, and when their initial remote consultations are conducted effectively, their trust and satisfaction levels increase markedly. Educational campaigns aimed at raising awareness and addressing common misconceptions have proven instrumental in fostering more favorable perceptions, thereby facilitating broader adoption of telemedicine as a viable complement to traditional dental care [35,37].

Moreover, the lack of clear legislative regulations and the absence of coherent reimbursement policies contribute to legal uncertainty and discourage individual initiatives [6]. In Romania, the absence of specific protocols and a comprehensive legal framework dedicated to teledentistry—highlighted by 27.4% of the study participants—contributes to a fragmented and inconsistent implementation of telemedicine within dental practice. This regulatory gap creates uncertainties regarding professional responsibilities, data protection requirements, and standards of care, which may deter both practitioners and patients from fully embracing teledentistry solutions. Such challenges are not unique to Romania; similar issues have been documented in other healthcare systems worldwide, where the lack of clear guidelines and legislative support impedes the standardization and wider adoption of telemedicine technologies in dentistry [6]. Addressing these regulatory deficiencies is essential to establish a secure, legally compliant environment that fosters confidence among healthcare providers and patients alike, thereby facilitating the sustainable integration of teledentistry into routine clinical practice) [6]. As the frequency of telemedicine use increased and its areas of application diversified, dental practitioners more frequently encountered challenges such as inadequate technical infrastructure and the absence of clear legislative regulations. The state authorities should develop clear and comprehensive regulations governing telemedicine and teledentistry to ensure legal clarity, data protection, and secure digital practice. At the same time, investment in modern healthcare IT infrastructure and reliable connectivity is essential, with dedicated funding programs to support smaller dental practices in adopting new technologies [43,44]. Furthermore, the creation of reimbursement models and financial incentives would encourage broader adoption and long-term sustainability of digital health solutions, including teledentistry [45]. In order to achieve these objectives, it is essential to develop first a national strategy for the digitalization of the healthcare system, establishing clear directions for development, measurable objectives, and interinstitutional coordination mechanisms, following the example of countries such as Saudi Arabia, Qatar or United Kingdom, where similar initiatives have accelerated the integration of digital technologies and contributed to increased efficiency and accessibility of medical services [15,44].

In the current study association rule method was implemented to identify the most frequent association of utilization situations and most frequent association of barriers. Also, this method offered insights regarding the association between the different types of usage situations and various barriers encountered. Regarding the association of implementation barriers, the most frequently cited obstacle—difficulty in performing clinical examinations (B3)—is commonly found in combination with two other barriers: the lack of legislative regulations (B5) and medical staff resistance to change (B6). These three obstacles often appear together as triad within the study cohort. Another frequently observed triad includes B3, B5, and inadequate technical infrastructure (B1). This pattern underscores the crucial role of state authorities in facilitating the adoption of teledentistry in Romania by establishing a clear legislative framework and providing financial support for acquiring necessary equipment. When analyzing combinations of two or three barriers among physicians with less experience using teledentistry, medical staff resistance to change (B6) appears less frequently. Conversely, this barrier is more prominent in similar combinations among physicians who have never used teledentistry. This suggests that medical staff reluctance decreases as the utilization of teledentistry increases. Also, the results showed that barrier inability to perform a direct, in-person clinical examination (B3) occur in a wide range of contexts, in different combinations with use cases: monitoring of treatment progress (U2), postoperative patient counseling (U3) while barrier absence of explicit and comprehensive legislative frameworks governing telemedicine practice (B5) tends to occur not only in certain situations of utilization (monitoring of treatment progress (U2), SARS-CoV-2 pandemic (U4)), but often in contexts with barriers insufficient technical infrastructure (B1) and patient hesitancy or reluctance to engage with telemedicine (B2).

Addressing the second research question, several major barriers to the implementation of teledentistry were identified, primarily the inability to perform direct clinical examinations, practitioner reluctance, and insufficient infrastructure. These findings are consistent with international reports, emphasizing the procedural nature of dentistry as a limiting factor for telemedical applications.

Approximately half of the participants in our study (47.2%) expressed a willingness and readiness to engage in training programs related to telemedicine, underscoring a substantial potential for the adoption and integration of this technology into dental practice. This interest reflects notable professional openness toward digital innovation, as well as the critical importance of both theoretical and practical training in this relatively new field. The need for professional development courses, and even the incorporation of telemedicine into dental school curricula, is similarly emphasized in international literature [7,46].

The proportion of respondents willing to attend training courses in telemedicine rises significantly among those who have already used teledentistry, reaching 58%. More than half of these practitioners recognize that effective utilization of telemedicine necessitates participation in professional training programs and workshops to acquire the essential technical and practical skills, particularly in a field that is relatively new and still lacks comprehensive regulation. In contrast, 38.5% of dentists who have never used teledentistry consider professional training to be necessary. Ghai S. (2020) emphasizes that educating and training dental professionals in telemedicine is key to increasing both its acceptance and adoption rates [7].

A particularly relevant aspect is the impact of the COVID-19 pandemic on dental practice. A total of 45.6% of respondents reported that telemedicine and digital technologies significantly and positively influenced the delivery of medical care during that period. Indeed, the pandemic context accelerated the implementation of alternative remote communication solutions, demonstrating the usefulness of telemedicine in ensuring the continuity of medical care under crisis conditions, when physical access to healthcare services was severely restricted [19,47]. Among the major benefits of teledentistry highlighted during this period are facilitation of remote consultations, monitoring of treatment progress, initial patient triage, reduction of direct physical contact, and decreased risk of infection transmission. These advantages emphasized the added value of digital technologies in maintaining standards of care and safety for both patients and healthcare professionals [36,48].

Nevertheless, only 36% of the dentists participating in this study believe that telemedicine will play a major role in the future of the dental profession. This cautious stance reflects an understanding of the inherent limitations of telemedicine in dentistry—a specialty where direct patient contact and hands-on clinical interventions remain indispensable. While telemedicine can be effective in complementary areas such as counseling, prevention, or post-treatment monitoring, it cannot fully replace the complexity and specificity of in-person dental care.

Regarding the third research question, prior experience with teledentistry was found to significantly shape practitioners’ perceptions and willingness to engage in future digital training. These results highlight the importance of continuous professional education and structured training initiatives to enhance digital competence and foster broader acceptance of teledentistry within clinical practice.

This study has several limitations that should be taken into account when interpreting the results. First, the geographic focus on Sibiu County limits the generalizability of the findings to the national level. Additionally, the response rate of 31.2%, though acceptable for online surveys, means a significant portion of the target population remains unrepresented. Because participation was based on voluntary responses to email invitations, there is a potential selection bias, as dentists who are more familiar with or interested in digital technologies may have been more likely to participate. Furthermore, the absence of comparative data from other medical specialties or regions within Romania restricts the study’s ability to explore variations related to professional context or geographic location.

To overcome the geographic limitation, future research should include a broader sample of dentists from multiple regions across Romania to capture a more comprehensive national perspective. Increasing response rates could be achieved through mixed-method data collection approaches, such as combining online surveys with in-person or telephone interviews, to reduce selection bias and ensure more representative participation. Additionally, comparative studies involving other medical specialties and healthcare settings would provide valuable insights into the varying levels of telemedicine adoption and perceptions across different fields.

## 5. Conclusions

This study reveals a moderate level of adoption of telemedicine within dental practice in Sibiu County, underscoring both the significant potential and the inherent limitations of this technology in a medical specialty fundamentally reliant on direct, in-person interaction between clinician and patient. With a utilization rate of 44.6%, the findings position this investigation within an intermediate spectrum of telemedicine use, comparable to or slightly exceeding rates reported in similar international studies. This suggests a genuine openness among dental professionals toward the integration of remote healthcare services, albeit accompanied by cautious awareness of the challenges involved.

Despite the well-recognized constraint posed by the inability to perform comprehensive clinical examinations remotely, study participants demonstrate an understanding of the distinct advantages telemedicine offers, such as enhanced collaboration, patient monitoring, and continuity of care, especially under circumstances limiting physical access to services.

Addressing these challenges necessitates a multifaceted approach, including the development and implementation of a robust legislative framework tailored to teledentistry, alongside government-backed initiatives to provide financial support for the acquisition of requisite technological infrastructure. Additionally, targeted educational programs and professional training are critical to enhancing digital competence and reducing resistance among healthcare providers. Promotional efforts aimed at increasing awareness and acceptance of telemedicine will further facilitate its integration into routine dental practice over the medium and long term.

Future research should expand on these findings by assessing the adoption and utilization patterns of teledentistry across diverse geographic regions within Romania, employing longitudinal methodologies to capture temporal trends. Furthermore, investigations into patient perceptions, satisfaction, and the clinical effectiveness of telemedicine-driven interventions will be essential to comprehensively evaluate the impact and sustainability of this evolving mode of healthcare delivery in dentistry. In conclusion, this study offers valuable insights into the current state of telemedicine adoption in dental practice within Sibiu County, highlighting both the opportunities and challenges associated with its integration. Despite the recognized benefits and growing interest, significant barriers remain, especially regarding clinical limitations, infrastructure, and legislative support. With targeted professional training and supportive policy measures, telemedicine has the potential to become a meaningful adjunct to traditional dental care, contributing to improved accessibility and continuity of services, particularly in times of healthcare crises.

## Figures and Tables

**Table 1:** Analysis of the relationship between utilization, barriers and the practice settings, years of experience.

Code	All n (%)	Dental Clinic Size n (%)				Years of Experience n (%)				
		1–2	3–5	>5	*p*	<5	5–10	11–20	>20	*P*
Adoption And Utilization										
U1	51 (25.89)	22 (21.57)	13 (26.53)	16 (34.78)	0.235	5 (16.67)	12 (34.29)	19 (23.75)	15 (28.85)	0.385
U2	50 (25.38)	21 (20.59)	13 (26.53)	16 (34.78)	0.181	7 (23.33)	9 (25.71)	15 (18.75)	19 (36.54)	0.184
U3	52 (26.40)	24 (23.53)	11 (22.45)	17 (36.96)	0.177	6 (20.00)	8 (22.86)	20 (25.00)	18 (34.62)	0.433
U4	48 (24.37)	24 (23.53)	8 (16.33)	16 (34.78)	0.107	2 (6.67)	7 (20.00)	19 (23.75)	20 (38.46)	0.011
U5	32 (16.24)	14 (13.73)	6 (12.24)	12 (26.09)	0.115	6 (20.00)	10 (28.57)	8 (10.00)	8 (15.38)	0.088
U6	58 (29.44)	27 (26.47)	14 (28.57)	17 (36.96)	0.427	6 (20.00)	12 (34.29)	18 (22.50)	22 (42.31)	0.053
Barriers										
B1	59 (29.95)	35 (34.31)	10 (20.41)	14 (30.43)	0.217	12 (40.00)	7 (20.00)	24 (30.00)	16 (30.77)	0.375
B2	56 (28.43)	23 (22.55)	13 (26.53)	20 (43.48)	0.031	12 (40.00)	15 (42.86)	17 (21.25)	12 (23.08)	0.040
B3	141 (71.57)	68 (66.67)	36 (73.47)	37 (80.43)	0.216	23 (76.67)	25 (71.43)	49 (61.25)	44 (84.62)	0.030
B4	28 (14.21)	15 (14.71)	5 (10.20)	8 (17.39)	0.592	3 (10.00)	5 (14.29)	9 (11.25)	11 (21.15)	0.381
B5	54 (27.41)	21 (20.59)	13 (26.53)	20 (43.48)	0.015	10 (33.33)	12 (34.29)	17 (21.25)	15 (28.85)	0.401
B6	73 (37.06)	43 (42.16)	17 (34.69)	13 (28.26)	0.249	9 (30.00)	11 (31.43)	36 (45.00)	17 (32.69)	0.295

**Table 2:** Co-occurrence of use case and barriers for all respondents and stratified by respondents who used vs. respondents who did not use telemedicine technologies.

	Co-Occurrence of 2 Items Items (Support)			Co-Occurrence of 3 Items Items (Support)		
	All	Used	Not Used	All	Used	Not Used
Adoption And Utilization	U2, U6 (21.8)	U2, U6 (48.9)		U2, U3, U6 (17.3)	U2, U3, U6 (38.6)	
	U3, U6 (20.3)	U3, U6 (47.7)		U2, U4, U6 (14.2)	U2, U4, U6 (31.8)	
	U2, U3 (19.8)	U2, U3 (44.3)		U2, U3, U4 (14.2)	U2, U3, U4 (31.8)	
	U3, U4 (17.3)	U3, U4 (38.6)		U1, U2, U6 (13.7)	U1, U2, U6 (30.7)	
	U4, U6 (16.8)	U4, U6 (37.5)		U1, U2, U3 (13.7)	U1, U2, U3 (30.7)	
	U1, U2 (16.8)	U1, U2 (37.5)		U1, U2, U4 (13.7)	U1, U2, U4 (28.4)	
Barriers	B3, B5 (23.4)	B3, B5 (23.9)	B3, B6 (27.5)	B1, B3, B5 (9.6)	B1, B3, B5 (9.1)	B3, B5, B6 (12.8)
	B3, B6 (22.3)	B1, B3 (19.3)	B3, B5 (22.9)	B3, B5, B6 (9.1)	B3, B4, B5 (9.1)	B1, B2, B3 (10.1)
	B1, B3 (19.8)	B2, B3 (19.3)	B1, B3 (20.2)	B3, B4, B5 (9.1)	B2, B3, B5 (8.0)	B1, B3, B5 (10.1)
	B2, B3 (19.8)	B3, B6 (15.9)	B2, B3 (20.2)	B1, B2, B3 (8.6)	B1, B2, B3 (6.8)	B1, B3, B6 (9.2)
	B1, B2 (13.2)	B3, B4 (13.6)	B5, B6 (12.8)	B2, B3, B5 (7.6)	B1, B2, B5 (6.8)	B3, B4, B5 (9.2)
	B3, B4 (12.7)	B1, B2 (13.6)	B3, B6 (12.8)	B1, B2, B6 (7.1)	B2, B3, B4 (5.7)	B2, B3, B6 (8.3)

## Data Availability

The data presented in this study are available on request from the corresponding author.

## Abbreviations

The following abbreviations are used in this manuscript:

U1	patient consultations
U2	monitoring of treatment progress
U3	postoperative patient counseling
U4	SARS-CoV-2 pandemic
U5	educational and preventive oral health
U6	consultations/collaboration with other dental specialists
B1	insufficient technical infrastructure
B2	patient hesitancy or reluctance to engage with telemedicine
B3	inability to perform a direct, in-person clinical examination
B4	concerns related to data confidentiality and information security
B6	resistance to change among medical personnel
ARM	association rule mining

## Appendix A

**Questionnaire: Telemedicine in Dentistry**

**Section 1**

1. You are:
  General dentist;
  Specialist dentist.
2. Your age:
  20–30
  31–40
  41–50
  >50
3. Gender:
  Female
  Male
4. How long have you been practicing dentistry:
  <5 years;
  5–10 years;
  11–20 years;
  >20 years.
5. You practice this profession in:
  Urban area;
  Rural area.
6. In the dental office/clinic where you work, the total number of dentists is:
  1–2 practicing dentists;
  3–5 practicing dentists;
  more than 5 practicing dentists;

**Section 2**

7. Have you ever used telemedicine in your dental practice?
  Yes
  No
8. For what purposes have you used telemedicine? (select all that apply)
  Patient consultation;
  Monitoring treatment progress;
  Postoperative counselling;
  During the SARS-CoV-2 pandemic;
  Education and prevention for patients;
  Collaboration with other specialists;
9. On a scale from 1 to 5, how useful do you consider telemedicine in dentistry? (1 = not useful, 5 = very useful)
  1 = not useful;
  2
  3
  4
  5 = very useful.
10. What are the barriers to the implementation of telemedicine? (select all that apply)
  Lack of adequate technical infrastructure;
  Patients’ reluctance toward online consultations;
  Difficulty in accurately assessing dental issues without direct examination;
  Confidentiality and data security issues;
  Lack of clear regulations for telemedicine in dentistry;
  It does not fit the specific nature of our work.
11. Would you be interested in attending training courses on the use of telemedicine in dentistry?
  Yes
  No
  Maybe.
12. Do you believe telemedicine will play an important role in the future of our profession?
  Yes
  No
  I don’t know;
13. Do you believe digital technologies will play an important role in the future of our profession?
  Yes
  No
  I don’t know.
14. Do you believe that digital technologies and telemedicine played an important role in practicing dentistry during the SARS-CoV-2 pandemic?
  Yes
  No
  I don’t know.
